# Magnetic cobalt oxide supported organosilica-sulfonic acid as a powerful nanocatalyst for the synthesis of tetrahydrobenzo[a]xanthen-11-ones

**DOI:** 10.1038/s41598-023-41234-x

**Published:** 2023-08-29

**Authors:** Hakimeh Ardeshirfard, Dawood Elhamifar

**Affiliations:** Department of Chemistry, Yasouj University, Yasouj, 75918-74831 Iran

**Keywords:** Catalysis, Organic chemistry

## Abstract

A novel core–shell structured magnetic cobalt oxide supported organosilica-sulfonic acid (Co_3_O_4_@SiO_2_/OS-SO_3_H) nanocomposite is prepared through a low-cost, simple, and clean method. The characterization of Co_3_O_4_@SiO_2_/OS-SO_3_H was performed by using Fourier transform infrared (FT-IR) spectroscopy, thermal gravimetric analysis (TGA), powder X-ray diffraction (PXRD), energy dispersive X-ray (EDX) spectroscopy, scanning electron microscopy (SEM), vibrating sample magnetometer (VSM), and transmission electron microscopy (TEM). The TGA and FT-IR results illustrate the high stability of the designed nanocomposite. The SEM image showed a size of about 40 nm for the Co_3_O_4_@SiO_2_/OS-SO_3_H nanoparticles. Furthermore, according to the result of VSM analysis, the saturation magnetization of this nanocomposite was about 25 emu/g. This novel material was used as an efficient nanocatalyst for the synthesis of biologically active tetrahydrobenzo[a]xanthen-11-one derivatives. These products were obtained in high to excellent yields under green conditions. The recoverability and reusability of this catalyst were also investigated under applied conditions.

## Introduction

The growth of magnetic nanoparticles (MNPs) from technological and scientific viewpoints has provided a new approach for medical applications, biotechnology, data storage, solid sensors, electrochromic, solar adsorbents, and catalytic applications^1–11^. Among different magnetic nanoparticles, the cobalt oxide NPs are very interesting for researchers due to their unparalleled properties such as good performance, high specific surface area, easy synthesis, high thermal and mechanical stability, and easy magnetic separation^12–23^. So far, different methods such as combustion, sol–gel, co-precipitation, chemical pyrolysis, and reduction have been used to synthesize magnetic cobalt oxide NPs. Between these, the reduction method has received special attention because of the low cost and time saving^24–31^. Since cobalt oxide NPs are chemically very active, they are easily oxidized and also self-aggregated in the environment. To solve these problems, the surface of these nanoparticles is coated with organic and inorganic materials and/or bioactive substances such as carbon, silica, polymers, peptides, etc*.*^32–39^. Among these, silica is more attractive due to its special properties such as its optical and magnetic transparency, high biocompatibility, high thermal and chemical stability, and non-toxicity. Also, silica prevents the aggregation of NPs and increases their stability. In addition, due to the presence of the hydroxyl groups on the silica surface, various catalytic functional moieties can be immobilized on it to increase the stability and performance of the final catalysts^40–42^. Some of recently reports in this matter are Co_3_O_4_@SiO_2_@TiO_2_-Ag^43^, Fe_3_O_4_@SiO_2_@GO^44^, Co_3_O_4_@SiO_2_/carbon nanocomposite^45^, Co_3_O_4_@SiO_2_-nylon6^37^, Fe_3_O_4_@SiO_2_-supported IL/[Mo_6_O_19_]^46^ and Fe_3_O_4_@SiO_2_@(BuSO_3_H)_3_^47^.

In recent years, the use of sulfonic acid groups as surface modifiers of core–shell structured nanoparticles has been considered by researchers. These have been used as strong and recoverable catalysts in organic reactions. Especially, sulfonic acid functionalized magnetic nanocomposites have been more interesting due to their easy magnetic separation. Some of reports in this matter are (Fe_3_O_4_@γFe_2_O_3_-SO_3_H)^48^, (Fe_3_O_4_@TDI@TiO_2_-SO_3_H)^49^, (Fe_3_O_4_@PDA-SO_3_H)^50^, (Fe_3_O_4_@D-NH-(CH_2_)_4_-SO_3_H)^51^ (Fe_3_O_4_@NS-GO)^52^ and (Fe_3_O_4_@OS-SO_3_H)^53^.

On the other hand, one-step multicomponent reactions that lead to the synthesis of heterocyclic compounds are one of the most practical and important organic processes. Among oxygen-containing heterocyclic compounds, xanthene derivatives have different biological applications such as antiviral, antibacterial, inhibitory, and antitumor. Therefore, in recent years, the synthesis of xanthen-11-one compounds has been investigated by using various catalysts. Some of the recently reported catalysts in this matter are *p*-toluenesulfonic acid (pTSA)^54^, trityl chloride (TrCl)^55^, ZnO NPs^56^, zwitterionic-type ionic liquid (CDIPS)^57^ and CoFe_2_O_4_/OCMC/Cu (BDC)^58^.

It is also important to note that, in recent years, the use of metal and metal oxide-based heterogeneous catalysts in organic transformations has been developed^59–62^. However, some of these catalytic systems suffer from problems of non-recoverability of catalyst, harsh reaction conditions, and the use of toxic organic solvents.

In view of the above and according to our experience in the preparation of magnetic nanocatalysts, herein, for the first time, a novel core–shell structured magnetic cobalt oxide supported organosilica-sulfonic acid (Co_3_O_4_@SiO_2_/OS-SO_3_H) nanocomposite is successfully prepared through a simple method. This namomaterial contains the advantages of magnetic nanoparticles such as easy separation and also the advantages of heterogeneous catalysts such as easy recoverability. The Co_3_O_4_@SiO_2_/OS-SO_3_H nanocomposite was characterized by using TGA, FT-IR, VSM, SEM, TEM, PXRD, and EDX analyses. The catalytic efficiency of this material was studied in the synthesis of biologically active xanthenes giving the desired products in high to excellent yields.

## Experimental

### Synthesis of Co_3_O_4_@SiO_2_

Magnetic Co_3_O_4_ nanoparticles were firstly synthesized through a reduction procedure as follows: CoCl_2_.6H_2_O (1.32 g) was added in 25 mL absolute EtOH while stirring at room temperature (RT). Then, ethanol-dissolved pluronic P123 (0.6 g in 7 mL EtOH) was added to the above solution. After complete mixing, NaBH_4_ (1.47 g) was added and the resulted combination was stirred for 10 min at RT. The obtained material was magnetically separated and washed completely with warm EtOH and water to remove pluronic P123 and other impurities. The product was dried at 65 °C for 5 h and called magnetic cobalt oxide (Co_3_O_4_). For the preparation of Co_3_O_4_@SiO_2_, the Co_3_O_4_ NPs (1 g) were dispersed in ethanol (60 mL), while ammonia (5.3 mL, 60% wt%) was added drop-wise. Then, tetraethylorthosilicate (TEOS, 1 mL) was slowly added and the resulted mixture was stirred at RT for 16 h. Finally, the magnetic solid product was collected using a magnet, washed with water and ethanol, dried at 70 °C for 6 h, and called Co_3_O_4_@SiO_2_ nanocomposite.

### Preparation of Co_3_O_4_@SiO_2_/OS-SH

To prepare the Co_3_O_4_@SiO_2_/OS-SH MNPs, the Co_3_O_4_@SiO_2_ nanomaterial (0.5 g) was added to a solution containing water (12 mL) and ethanol (50 mL). The resulting mixture was stirred at RT for 30 min. Then, ammonia (2 mL, 25% wt%) was added and it was stirred at RT for another 10 min. Next, tetraethylorthosilicate (TEOS, 1 mL) and 1,2-bis(triethoxysilyl)methane (BTEM, 1 mL) were added drop-wise, and the obtained mixture was stirred at RT for 16 h. The product was magnetically separated, washed with absolute ethanol and water, and dried at 70 °C for 6 h. After that, the resulting material (1 g) was dispersed in dried toluene (25 mL), while (3-mercaptopropyl)trimethoxysilane (0.7 mmol) was added. This mixture was refluxed for 24 h. The final product was magnetically separated, washed with absolute ethanol and water, dried at 70 °C for 6 h, and denoted as Co_3_O_4_@SiO_2_/OS-SH.

### Preparation of Co_3_O_4_@SiO_2_/OS-SO_3_H

For this, Co_3_O_4_@SiO_2_/OS-SH (0.5 g) was first dispersed in methanol (20 mL). Then, hydrogen peroxide (5 mL, 35%) was added and the resulted mixture was stirred at ambient temperature for 24 h. The product was separated by using an external magnetic field. After that, this was added to a flask containing sulfuric acid solution (25 mL, 2 M) and stirred at RT for 3 h. The resulted material was separated, washed with ethanol and water, dried at 70 °C for 5 h, and denoted as Co_3_O_4_@SiO_2_/OS-SO_3_H.

### Synthesis of tetrahydrobenzo[a]xanthen-11-one derivatives

For this purpose, a mixture of benzaldehyde (1 mmol), dimedone (1 mmol), 2-naphthol (1 mmol), and Co_3_O_4_@SiO_2_-SO_3_H nanocatalyst (0.015 g) was stirred at 60 °C. The reaction progress was monitored by TLC. At the end of the reaction, the nanocatalyst was collected by a magnet and pure products were obtained after recrystallization in ethanol.

### Spectral data of xanthene products

#### 9,9-Dimethyl-12-phenyl-8,9,10,12- tetrahydrobenzo[a]xanthen-11-one

FT-IR (KBr, cm^-1^): 3058 (=CH, aromatic), 2932 (–CH, aliphatic), 1647 (C=O), 1616 (C=C, olefin), 1595, 1471 (C=C, aromatic), 1227 (C–O). ^1^H-NMR (400 MHz, DMSO, δ ppm): 0.97 (s, 3H), 1.13 (s, 3H), 2.26 (d, J = 16 Hz, 1H, COCH2), 2.31 (d, J = 16.4 Hz, 1H, COCH2), 2.58 (s, 2H), 5.72 (s, 1H), 7.07 (t, J = 7.6, 1H), 7.19 (t, J = 8, 2H), 7.33–7.47 (m, 5H), 7.76 (d, J = 8.4 Hz, 1H), 7.78 (d, J = 6.4 Hz, 1H), 8.00 (d, J = 8.4 Hz, 1H). ^13^C-NMR (100 MHz, DMSO, δ ppm): 28.40, 33.2, 34.0, 40.6, 51.3, 117.7, 119.7, 123.9, 124.2, 125.3, 127.3, 127.6, 128.0, 128.2, 128.9, 129.2, 130.5, 132.6, 142.2, 154.3, 164.2, 197.5.

#### 9,9-Dimethyl-12-(4-nitrophenyl)-8,9,10,12-tetrahydrobenzo[a]xanthen-11-one

FT-IR (KBr, cm^-1^): 3060 (=CH, aromatic), 2930 (–CH, aliphatic), 1650 (C=O), 1618 (C=C, olefin), 1594, 1475 (C=C, aromatic), 1515, 1341 (NO_2_), 1223 (C–O). ^1^H-NMR (400 MHz, DMSO, δ ppm): 0.98 (s, 3H), 1.00 (s, 3H), 2.24 (d, J = 16.5 Hz, 1H, COCH2), 2.35 (d, J = 16 Hz, 1H, COCH2), 2.60 (s, 2H), 5.69 (s,1H), 7.34 (d, J = 9.2, 1H), 7.38–7.48 (m, 2H), 7.51 (d, J = 8.8 Hz, 2H), 7.82–7.85 (m, 3H), 8.06 (d, J = 8.5 Hz, 2H). ^13^C-NMR (100 MHz, DMSO, δ ppm): 28.2, 33.2, 33.9, 40.5, 51.4, 117.8, 119.8, 123.8, 124.1, 125.5, 127.6, 128.0, 129.1, 129.2, 130.5, 132.6, 147.1, 147.3, 154.2, 164.1, 197.4.

## Results and discussion

**Figure 1:**
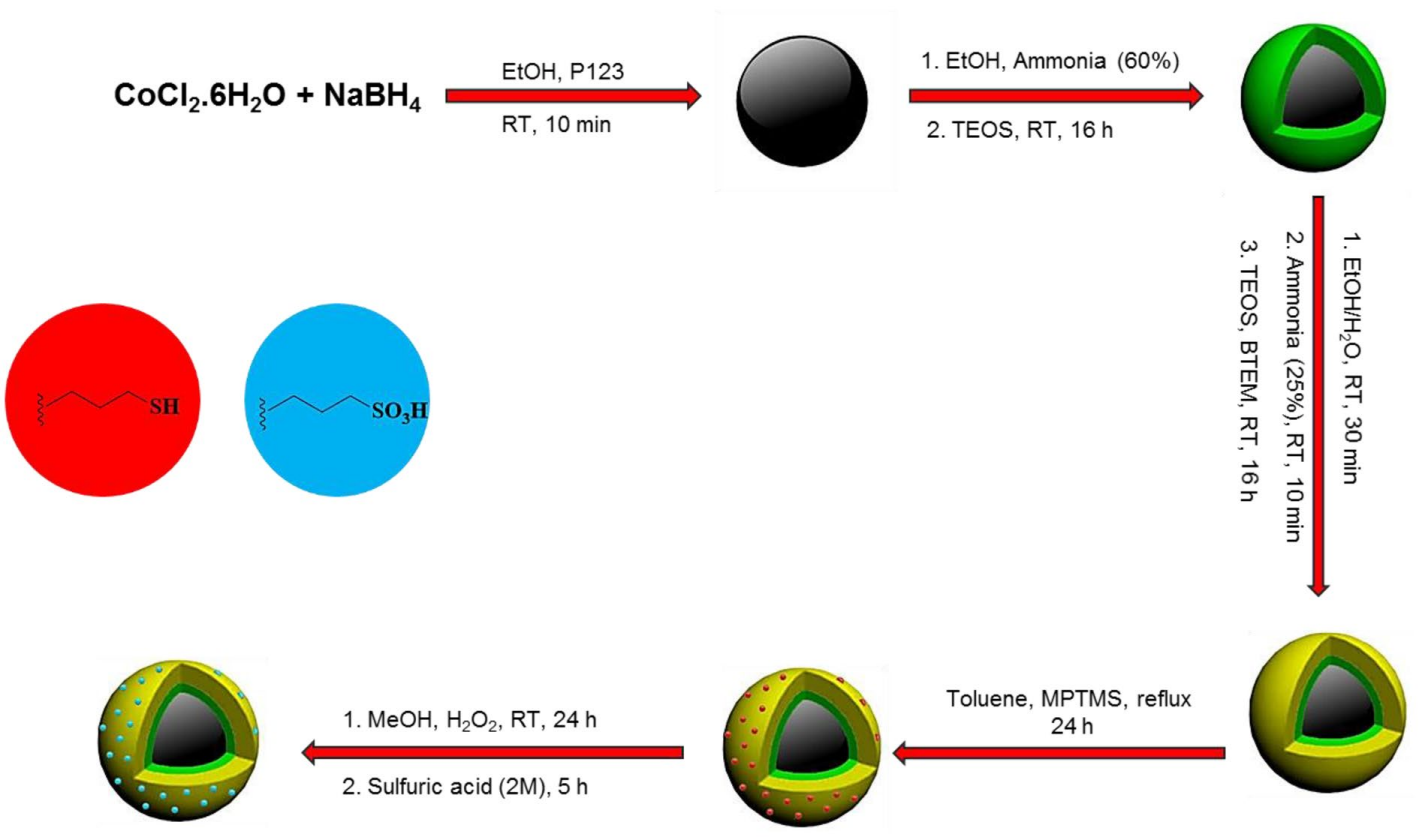
Preparation of Co_3_O_4_@SiO_2_/OS-SO_3_H.

The Co_3_O_4_ NPs were synthesized through the reduction of CoCl_2_.6H_2_O in the presence of NaBH_4_. Next, Co_3_O_4_@SiO_2_ nanocomposite was prepared via a sol–gel process. In the following, the surface of Co_3_O_4_@SiO_2_ was modified with a layer of organosilica through co-condensation of BTEM and TEOS to give Co_3_O_4_@SiO_2_/OS nanomaterial. In fact, the SiO_2_/OS shell was prepared to protect Co_3_O_4_ NPs against oxidation and destruction by acid. Moreover, the organosilica (OS) layer also increases the surface lipophilicity of the material improving the performance of the designed catalyst in organic reactions. After that, Co_3_O_4_@SiO_2_/OS was modified with MPTMS groups to deliver Co_3_O_4_@SiO_2_/OS-SH nanocomposite. Finally, to obtain the Co_3_O_4_@SiO_2_/OS-SO_3_H nanocatalyst, the SH moieties of the Co_3_O_4_@SiO_2_/OS-SH nanocomposite were oxidized to SO_3_H in the presence of H_2_O_2_ (Fig. 1).

**Figure 2:**
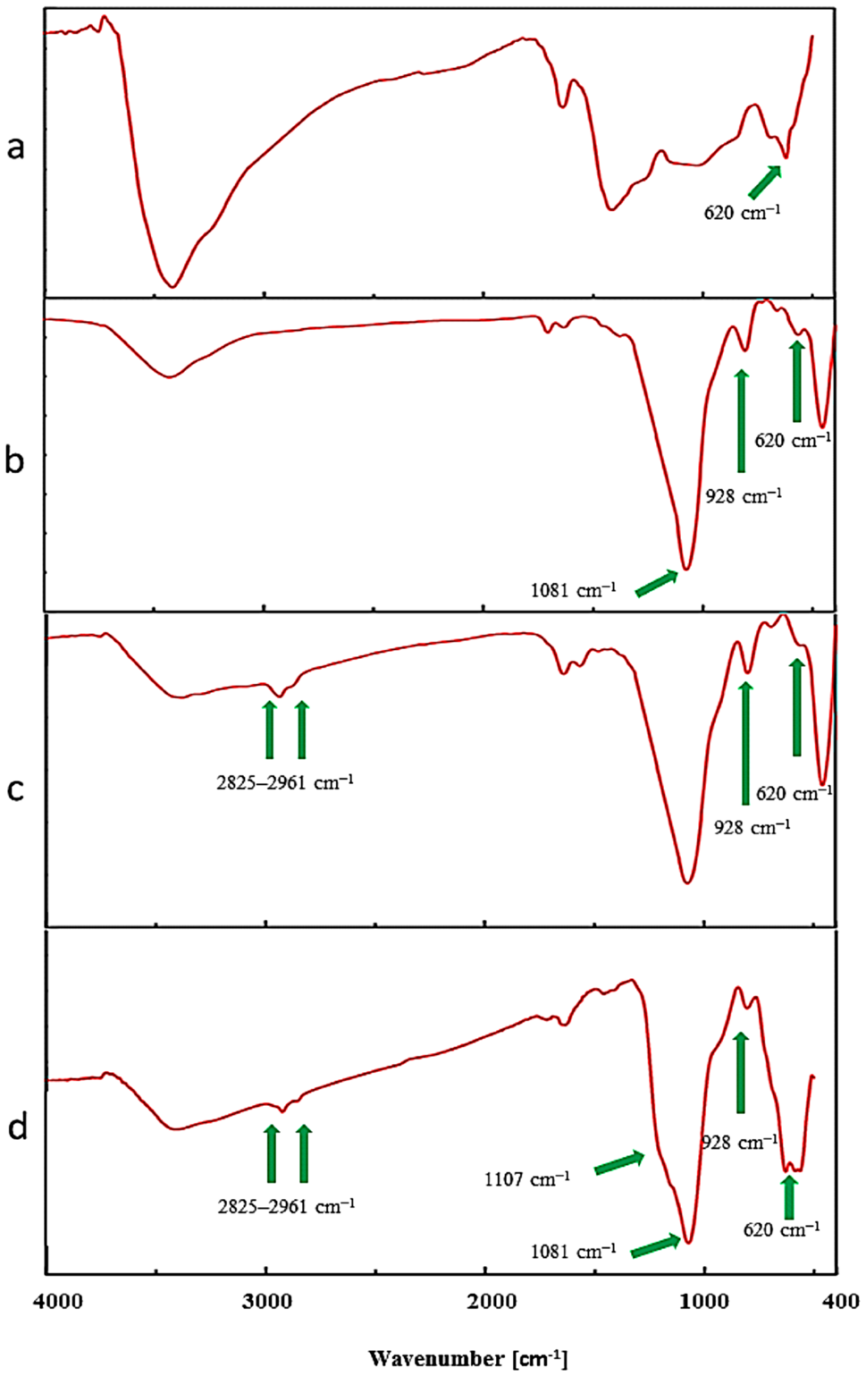
FT-IR spectra of (**a**) Co_3_O_4_, (**b**) Co_3_O_4_@SiO_2_, (**c**) Co_3_O_4_@SiO_2_/OS and (**d**) Co_3_O_4_@SiO_2_/OS-SO_3_H nanomaterials.

Figure 2 shows the FT-IR spectra of Co_3_O_4_, Co_3_O_4_@SiO_2_, Co_3_O_4_@SiO_2_/OS, and Co_3_O_4_@SiO_2_/OS-SO_3_H. The characteristic peaks at 3400 and 620 cm^−1^, for all materials, are related to the O–H and Co–O bonds, respectively (Figs. 2a–d). The intense absorption peaks at 1081 and 928 cm^−1^ are, respectively, related to unsymmetrical and symmetrical vibrations of the Si–O–Si bonds (Figs. 2b–d). Also, the peaks observed at 2825–2961 cm^−1^ can be assigned to the vibrations of aliphatic C–H bonds (Figs. 2c,d). The peak observed at 1107 cm^−1^ is related to S=O bond, which is partially overlapped with the silica peaks, confirming the successful oxidation of SH–SO_3_H (Fig. 2d).

**Figure 3:**
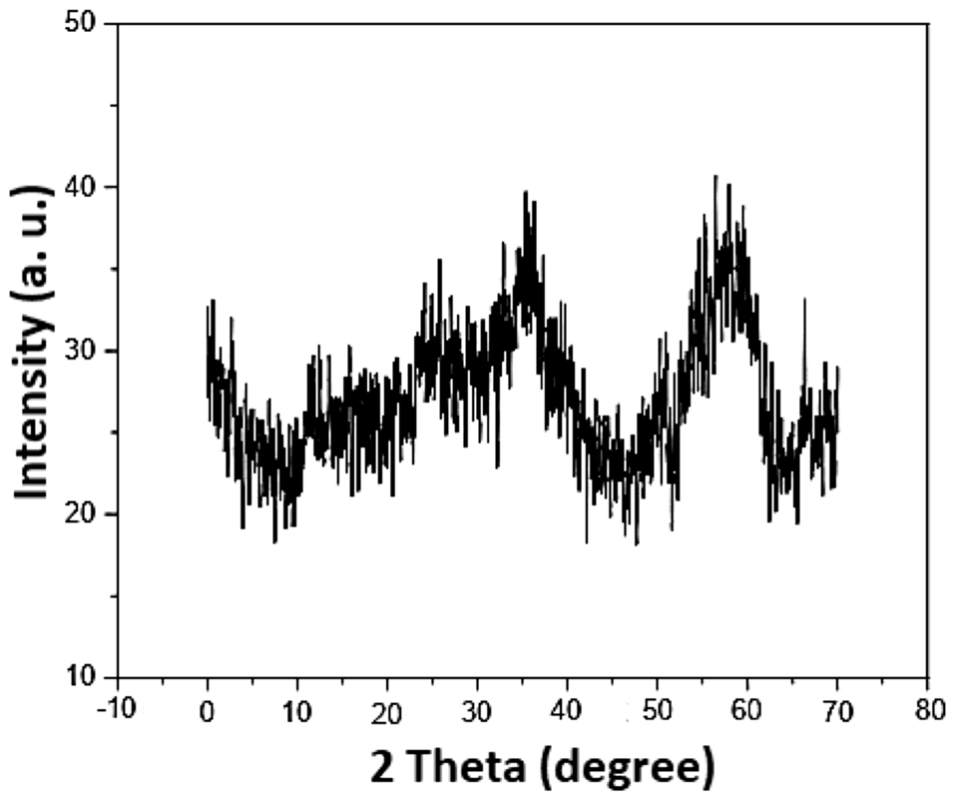
PXRD pattern of Co_3_O_4_@SiO_2_/OS-SO_3_H.

Figure 3 illustrates the PXRD pattern of Co_3_O_4_@SiO_2_/OS-SO_3_H nanocomposite. As shown, the diffraction peaks of Co_3_O_4_ NPs are appeared at 2θ = 23.2°, 30.1°, 35.5°, 41.2°, 47.5°, 60° and 71.6° proving the high stability of crystalline structure of the Co_3_O_4_ NPs during the synthesis of Co_3_O_4_@SiO_2_/OS-SO_3_H nanocomposite.

**Figure 4:**
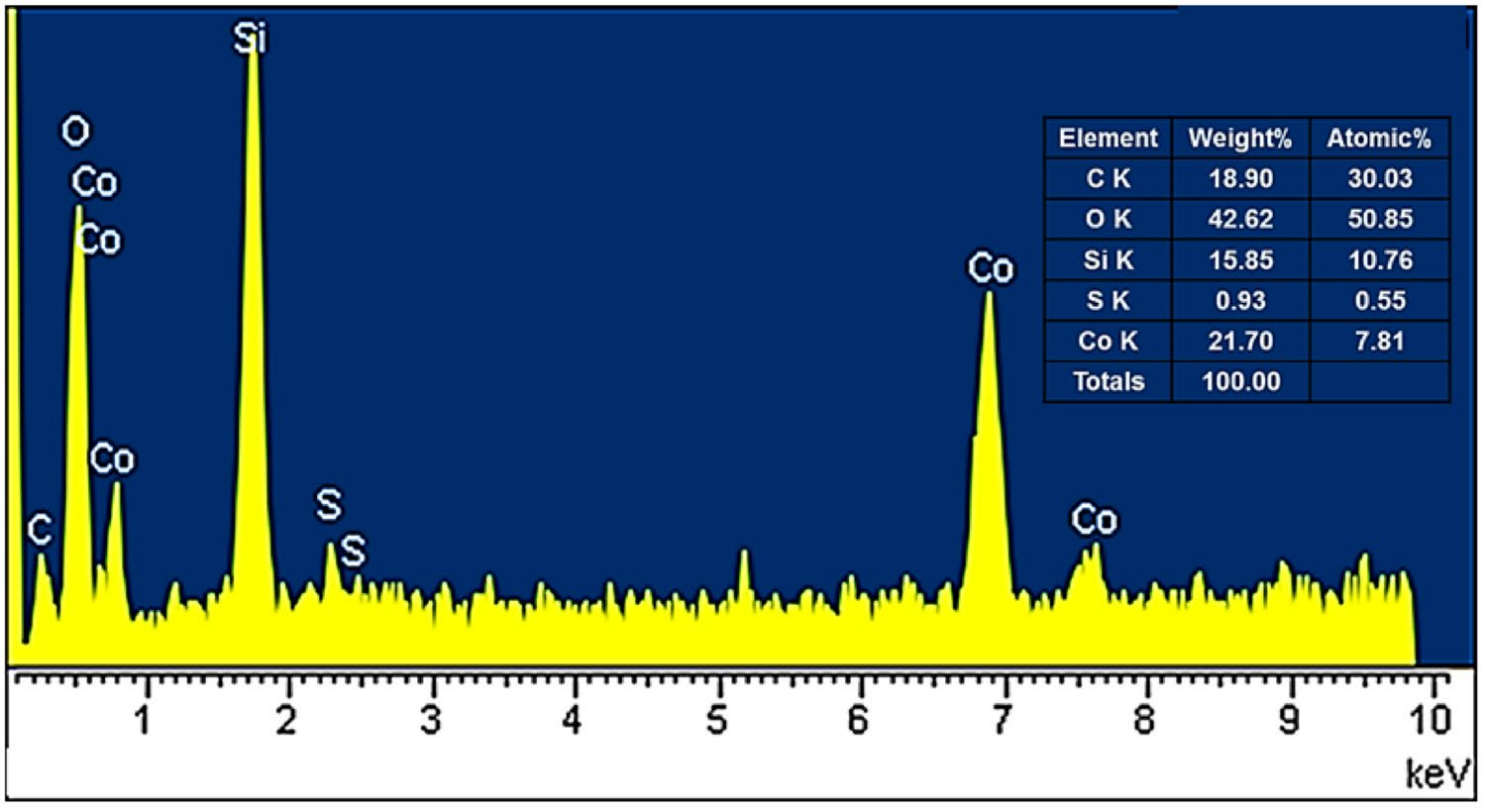
EDX spectrum of Co_3_O_4_@SiO_2_/OS-SO_3_H.

The EDX analysis was used to investigate the presence of O, C, Co, Si, and S in the structure of the Co_3_O_4_@SiO_2_/OS-SO_3_H nanocomposite. As shown in Fig. 4, the signals of C, O, Si, S and Co elements are clearly seen in weight% of 18.9, 42.62, 15.85, 0.93, 21.7 and 21.7, respectively. This confirms the successful incorporation/immobilization of cobalt oxide and silicasulfonic acid moieties into/onto the framework of the designed nanocomposite.

**Figure 5:**
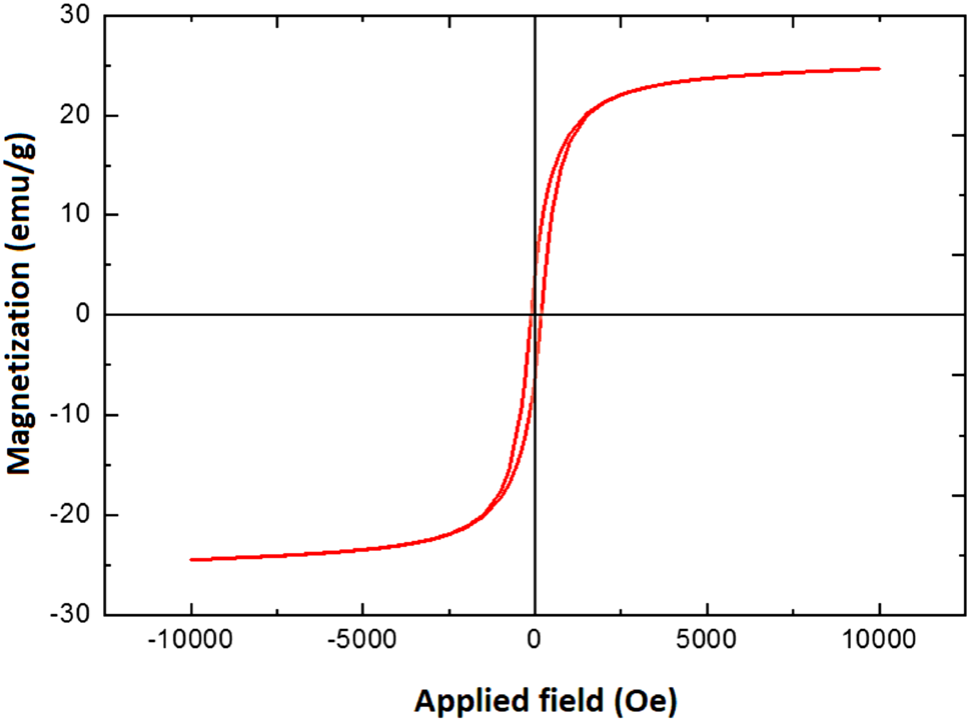
VSM analysis of Co_3_O_4_@SiO_2_/OS-SO_3_H.

Also, the VSM analysis of the Co_3_O_4_@SiO_2_/OS-SO_3_H nanocomposite showed a saturation magnetization of about 25 emu/g (Fig. 5). This result proves the good magnetic properties of the prepared nanocomposite which is a very important characteristic in the catalytic processes.

**Figure 6:**
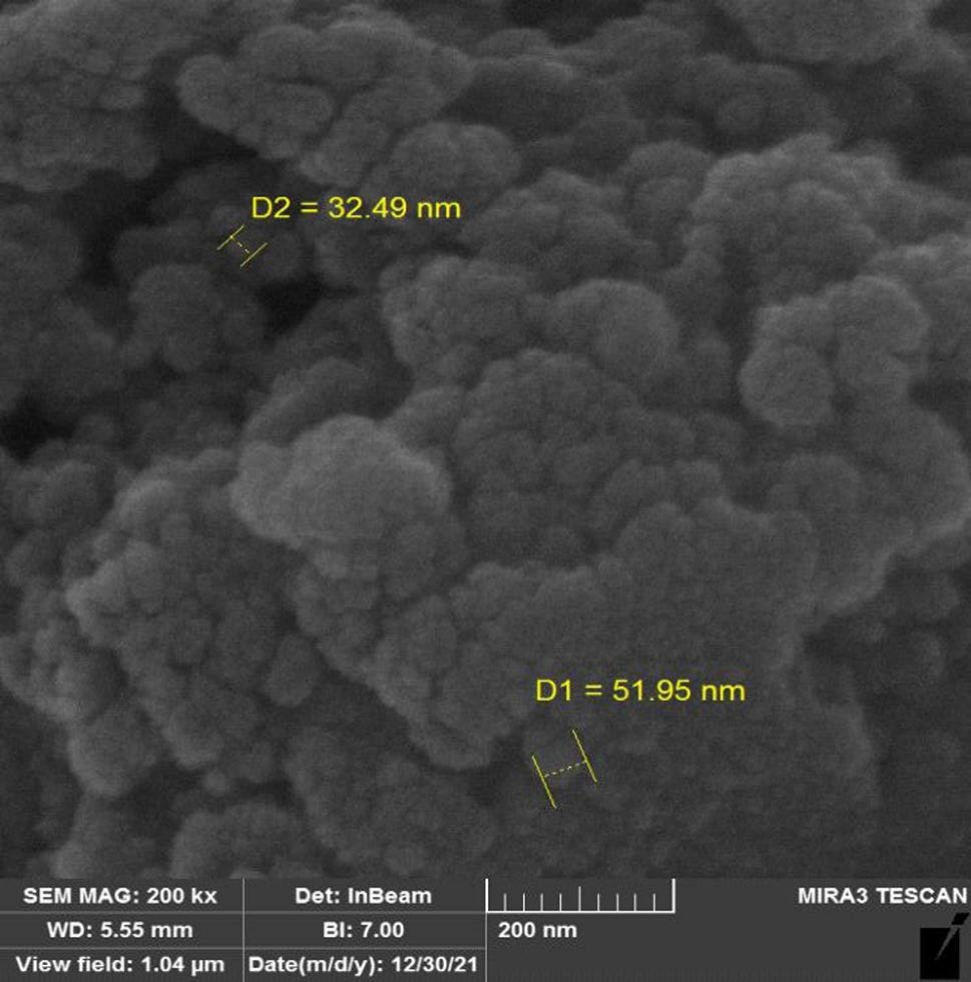
SEM image of Co_3_O_4_@SiO_2_/OS-SO_3_H.

The SEM image of the Co_3_O_4_@SiO_2_/OS-SO_3_H nanocomposite is demonstrated in Fig. 6. As shown, sponge-like particles with spherical morphology and an average size of 40 nm are observed for this material.

**Figure 7:**
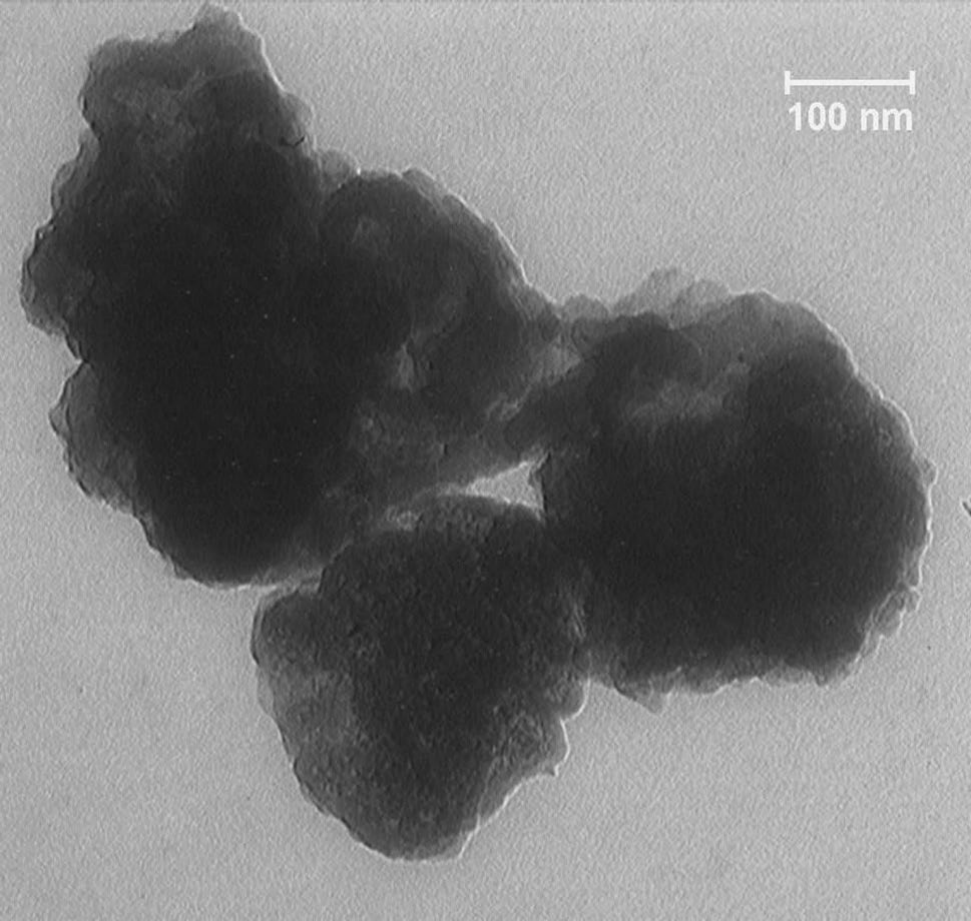
TEM image of Co_3_O_4_@SiO_2_/OS-SO_3_H.

TEM image of Co_3_O_4_@SiO_2_/OS-SO_3_H nanocomposite confirmed that the structure of the designed nanocomposite is almost spherical. Also, the image demonstrates a dark core (Co_3_O_4_) enclosed by a gray silica/organosilica layer confirming the core shell structure of this nanocomposite (Fig. 7).

**Figure 8:**
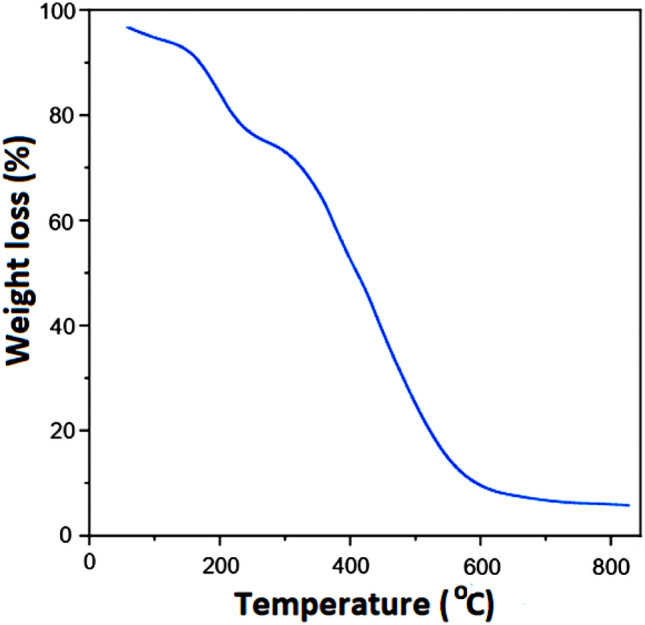
TG analysis of Co_3_O_4_@SiO_2_/OS-SO_3_H.

Thermal stability of the prepared nanocomposite was also investigated by thermal gravimetric analysis (TGA). The first weight loss below 150 °C is corresponded to the removal of adsorbed water and alcoholic solvents. The second weight loss at 151–220 °C is related to elimination of supported propansulfonic acid moieties. The main weight loss observed at 225–600 °C is corresponded to decomposition and removal of organic groups in the shell framework. These results confirm high thermal stability of the designed catalyst (Fig. 8).

After the characterization of the designed nanocomposite, its catalytic activity was studied in the synthesis of tetrahydrobenzo[a]xanthen-11-ones. To optimization of the conditions, the condensation between benzaldehyde, dimedone, and 2-naphthol was selected as a test model. The study showed that the presence of the catalyst is necessary for the progress of the reaction and by using 0.015 g of Co_3_O_4_@SiO_2_/OS-SO_3_H, the highest yield was resulted (Table 1, entries 1–4). The study also showed that among EtOH, toluene, CH_2_Cl_2_, and CH_3_CN solvents, in EtOH the best result is obtained (Table 1, entry 3 vs. entries 5–7). Also, the study of the temperature effect illustrated that the best temperature for this process is 60 °C (Table 1, entry 3 vs. entries 8–11). Accordingly, the use of 0.015 g catalyst, ethanol solvent, and 60 °C were selected as optimum conditions (Table 1, entry 3).

**Table 1:** The effect of different parameters in the synthesis of tetrahydrobenzo[a]xanthen-11-one.

Entry	Catalyst (g)	Solvent	T (°C)	Yield (%)
1	–	EtOH	60	–
2	0.01	EtOH	60	82
3	0.015	EtOH	60	95
4	0.02	EtOH	60	95
5	0.015	Toluene	60	27
6	0.015	CH_2_Cl_2_	60	47
7	0.015	CH_3_CN	60	58
8	0.015	EtOH	25	27
9	0.015	EtOH	35	54
10	0.015	EtOH	50	78
11	0.015	EtOH	70	95

Next, the substrate scope of this catalytic system was studied under optimal conditions. This demonstrated that all aldehyde substrates containing both electron-donor and electron-acceptor substituents successfully react with dimedone and 2-naphthol in the presence of Co_3_O_4_@SiO_2_/OS-SO_3_H nanocatalyst to give corresponding tetrahydrobenzo[a]xanthen-11-one in high yields (Table 2).

**Table 2:** Synthesis of tetrahydrobenzo[a]xanthen-11-ones in the presence of Co_3_O_4_@SiO_2_/OS-SO_3_H.

Entry	Aldehyde	Time (min)	Yield (%)	M. P. (°C)	
				Found	Reported
1	C_6_H_5_	40	95	150–151	152–154^63^
2	4-NO_2_-C_6_H_5_	35	97	182–184	180–181^63^
3	4-Br-C_6_H_5_	35	95	183–185	185–187^55^
4	2,4-Cl-C_6_H_5_	45	91	180–182	179–181^55^
5	2-NO_2_-C_6_H_5_	40	90	223–225	221–224^64^
6	2-Cl-C_6_H_5_	35	95	173–175	174–176^64^
7	4-MeO-C_6_H_5_	50	92	199–202	201–203^65^
8	4-HO-C_6_H_5_	50	90	221–222	220–225^65^

Then, the recoverability and reusability of the Co_3_O_4_@SiO_2_/OS-SO_3_H nanocatalyst were investigated. For this, the reaction between benzaldehyde, dimedone, and 2-naphthol by using Co_3_O_4_@SiO_2_/OS-SO_3_H nanocatalyst under optimal conditions was selected as a test model. After completion of the process, Co_3_O_4_@SiO_2_/OS-SO_3_H was separated and reused in another reaction under the same conditions as the first run. These steps were repeated and the results displayed that Co_3_O_4_@SiO_2_/OS-SO_3_H can be recovered and reused for at least seven runs with no important decrease in its activity (Table 3).

**Table 3:** Recoverability and reusability of the Co_3_O_4_@SiO_2_/OS-SO_3_H nanocatalyst.

Run	Time (min)	Yield (%)	Run	Time (min)	Yield (%)
1	40	95	5	45	93
2	40	95	6	50	91
3	40	95	7	55	87
4	45	95	8	65	84

To prove the stability of the catalyst structure during the reaction, the recovered catalyst, after the fifth run, was washed several times with ethanol and characterized by using EDX and PXRD analyses. The PXRD analysis of the recovered catalyst showed a pattern with seven peaks at 2θ = 23.2°, 30.1°, 35.5°, 41.2°, 47.5°, 60° and 71.6° (SI, Fig. 1S). This result is in good agreement with the PXRD pattern of the fresh nanocatalyst confirming the high stability of the crystalline structure of magnetic Co_3_O_4_@SiO_2_/OS-SO_3_H NPs under applied conditions.

The EDX analysis of the recovered catalyst, after the fifth run, also showed the presence of expected C, O, Si, S, and Co elements in a wt% of 18.65, 42.18, 15.71, 0.9, and 22.56, respectively (SI, Fig. 2S). These results are approximately the same as those of fresh catalyst confirming the high stability of the composition of the designed catalyst under applied conditions.

The activity of Co_3_O_4_@SiO_2_/OS-SO_3_H nanocatalyst was compared with a number of catalysts reported in the synthesis of tetrahydrobenzo[a]xanthen-11-ones (Table 4). The result showed that our catalyst is better than others in terms of recovery times, reaction temperature, and yield of product. These findings can be ascribed to high stability, good lipophilicity, and the magnetic properties of the Co_3_O_4_@SiO_2_/OS-SO_3_H nanocatalyst.

**Table 4:** Comparison the efficiency of the Co_3_O_4_@SiO_2_/OS-SO_3_H nanocatalyst with that of formerly reported catalytic systems in the synthesis of tetrahydrobenzo[a]xanthen-11-ones.

Entry	Catalyst	Conditions	Recovery times	Time (min)	Yield (%)	References
1	NH_2_SO_3_H	[BMIM]BF_4_, 80 °C	4	60	85	^66^
2	Fe_3_O_4_@nano-walnut shell/B^III^ (FNWSB)	Solvent-free, 80 °C	4	40	92	^67^
3	Fe_3_O_4_@chitosan	EtOH, 40 °C	4	180	90	^68^
4	Cu/Fe_3_O_4_@APTMS-DFX	Solvent-free, 120 °C	5	45	90	^69^
5	KF/CP NPs	Solvent-free, 80 °C	4	60	91	^70^
6	Co_3_O_4_@SiO_2_/OS-SO_3_H	EtOH, 60 °C	7	40	95	This work

**Figure 9:**
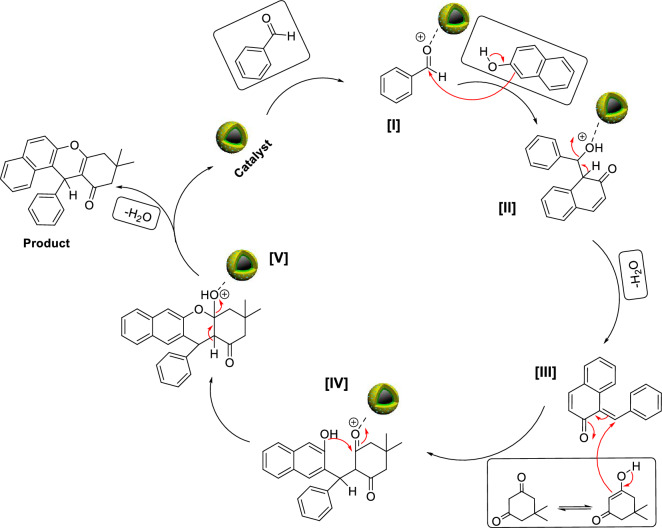
Plausible mechanism for the synthesis of tetrahydrobenzo[a]xanthen-11-ones in the presence of Co_3_O_4_@SiO_2_/OS-SO_3_H.

A plausible mechanism for the synthesis of tetrahydrobenzo[a]xanthen-11-ones in the presence of Co_3_O_4_@SiO_2_/OS-SO_3_H is shown in Fig. 9. Firstly, the aldehyde is activated by the catalyst to give intermediate [I]. Next, the activated aldehyde and 2-naphthol react with each other via a Knoevenagel condensation to deliver intermediate [II]. In the next step, the nucleophilic attack of dimedone to the intermediate [II], through a Michael-type addition, gives intermediate [IV]. Finally, an intramolecular cyclization is performed to give the desired product.

## Conclusion

In summary, a novel core–shell structured magnetic cobalt oxide supported organosilica-sulfonic acid nanocomposite was synthesized and called Co_3_O_4_@SiO_2_/OS-SO_3_H. The high thermal and chemical stability of the designed nanocomposite were confirmed by using EDX, TGA and FT-IR techniques. The SEM and TEM images illustrated a spherical morphology for this material. The good magnetic property of this material was confirmed by VSM. The Co_3_O_4_@SiO_2_/OS-SO_3_H nanocomposite was used as an effective catalyst in the synthesis of tetrahydrobenzo[a]xanthen-11-ones under mild reaction conditions. The desired xanthene products were obtained in high to excellent yield and selectivity at a relatively short reaction time. The catalyst was also recovered and reused several times with no significant decrease in its efficiency. Some applications of Co_3_O_4_@SiO_2_/OS-SO_3_H in other chemical processes are underway in our laboratory.

### Supplementary Information

Supplementary Figures.

## Data Availability

All data and materials are included in the manuscript.
