# Supplementary material for: Magnetic cobalt oxide supported organosilica-sulfonic acid as a powerful nanocatalyst for the synthesis of tetrahydrobenzo[a]xanthen-11-ones
Source: Sci Rep. 2023 Aug 29;13:14134. doi: 10.1038/s41598-023-41234-x (PMC10465481; doi:10.1038/s41598-023-41234-x)
Supplement: Supplementary file 1 — Supplementary Figures. [file 41598_2023_41234_MOESM1_ESM.pdf]

Supporting Information for:

Magnetic cobalt oxide supported organosilicaulfonic acid as a powerful nanocatalyst for the synthesis of tetrahydrobenzo[*a*]xanthen-11-ones

Hakimeh Ardeshirfard and Dawood Elhamifar*

Department of Chemistry, Yasouj University, Yasouj 75918-74831, Iran

Email: d.elhamifar@yu.ac.ir

Contents:

Figure 1S. PXRD pattern of the recovered Co_3O_4@SiO_2/OS-SO_3H nanocatalyst

Figure 2S. EDX analysis of the recovered Co_3O_4@SiO_2/OS-SO3H nanocatalyst

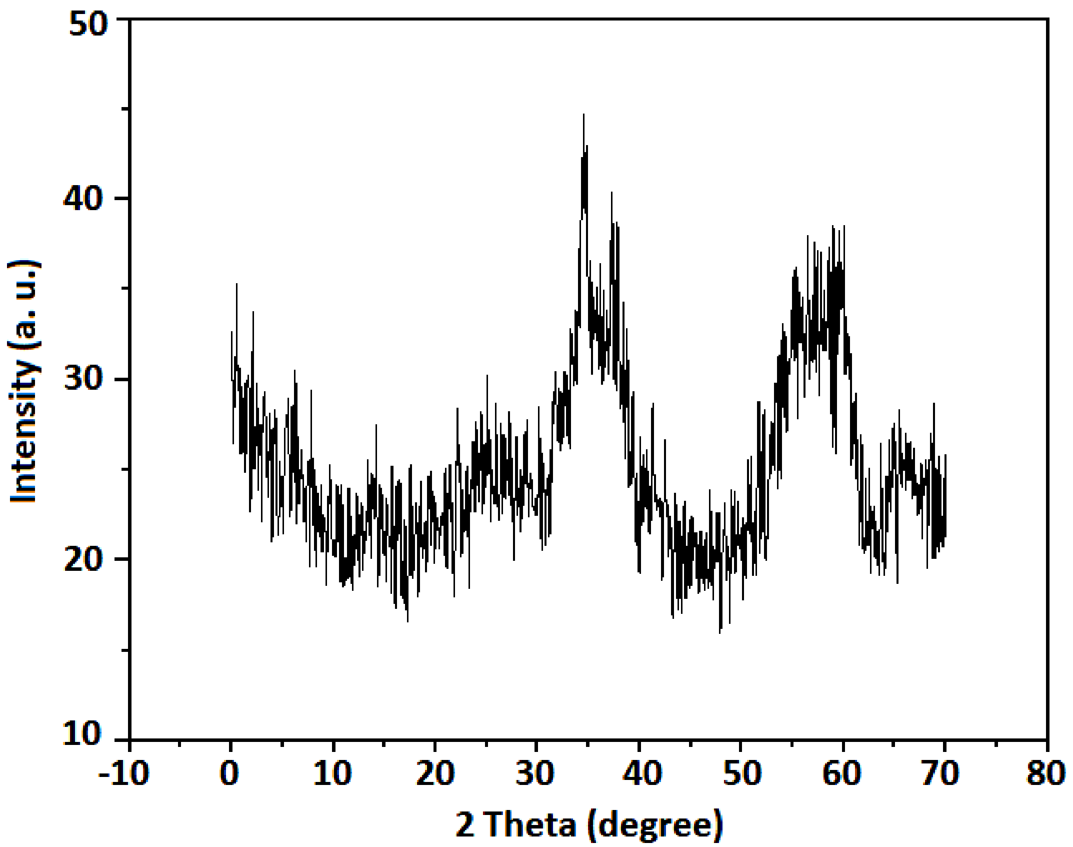

Figure 1S. PXRD pattern of the recovered Co_3O_4@SiO_2/OS-SO_3H nanocatalyst

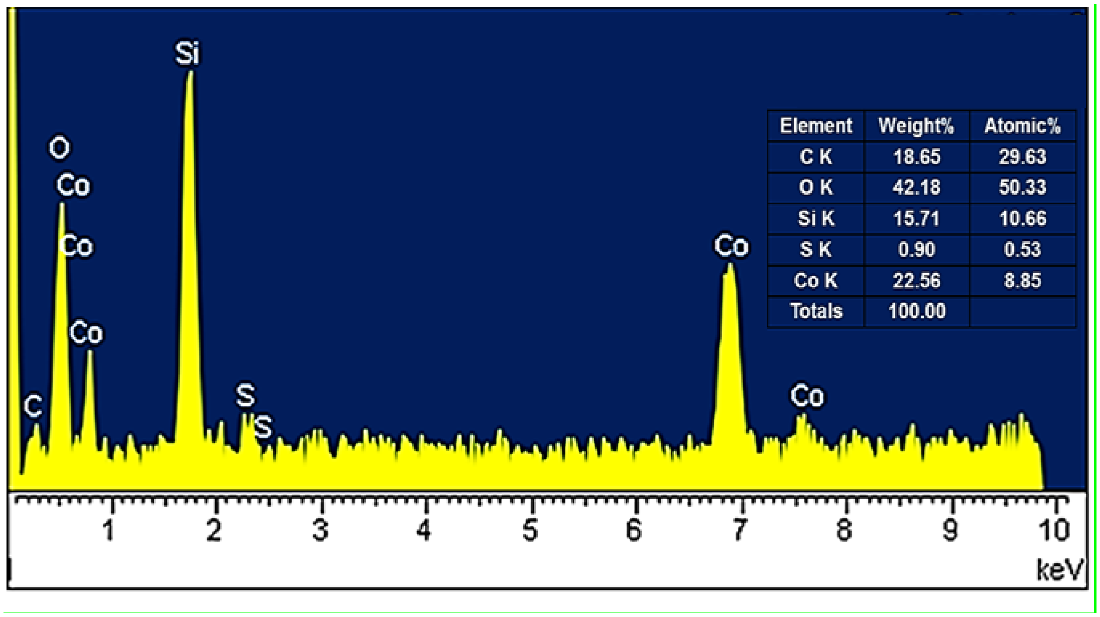

Figure 2S. EDX analysis of the recovered Co_3O_4@SiO_2/OS-SO_3H nanocatalyst
